# A Disease of the Skin Occurring on the Hands of Workers among Sheep

**Published:** 1908-08-01

**Authors:** Howard G. Pesel


### The General Practitioner's Column.

[Contributions to this Column are invited, and if accepted will be paid for.]

### A DISEASE OF THE SKIN OCCURRING ON THE HANDS OF WORKERS AMONG SHEEP.

By HOWARD G. PESEL, M.D.

The following account of a skin disease, which the writer has not previously seen described, may be of interest:—

The first case which came under his notice was that of a farmer who presented himself in May, 1907, complaining of a painful swelling on the wrist and pain shooting up his arm. He had recently been working among his sheep. In appearance the tumour was about the size of a shilling, with a purple centre and œdematous margin, and was hard on palpation. The general appearance was distinctly suggestive of malignant pustule. Antiseptic fomentations were applied.

Next morning the tumour had increased in size and was more painful; so in view of the suspicious nature of the swelling it was excised under local anæsthesia. It felt tough under the knife and was œdematous at its base.

The wound healed readily, and there was no recurrence. The excised portion was sent away for microscopic examination. The report said that no trace of anthrax could be found, but the fluid which exuded contained a bacillus resembling that of malignant œdema. A similar case similarly treated, that of a farm foreman, occurred in the same month in the practice of the writer's partner.

In May, 1908, two patients came to the surgery with the same complaint. They had both been dressing the same flock of sheep for "thrush"—a warty disease of the lips and mouth. The first had only one small nodule on the knuckles. There was pain in the lump and up the arm. Some serum was evacuated and a detergent coal-tar lotion given. The tumour disappeared in about a week, pain persisting for the first four days. In the other there was a large purple lump on the proximal phalanx of one little finger with a bleb surrounding it and covering two-thirds of the circumference of the finger. There was another similar, though smaller, lump on the knuckles, and a third on the palmar surface of the wrist. The bleb on the little finger was incised and a quantity of serum evacuated. Fomentations of a coal tar lotion were ordered to be used as frequently as possible. Next day the pain was still severe and had kept the patient awake at night. The tumours were incised to let out the contained serum. The pain gradually subsided and the tumours disappeared within a fortnight. There was a sharp dermatitis due to the lotion, but this quickly yielded to treatment.

Three points are noticeable in the complaints. In all the cases it occurred on the hands and wrists. In all the month was May, and all the patients had worked among sheep.

It has been suggested by a medical neighbour that the trouble might be due to arsenic in the lotion used for dressing the "thrush," but the veterinary surgeon in charge of the flock says that there is no arsenic in the lotion, and that the active ingredient is borax.

Some tar preparation has always been found most effective in treatment, but it must be vigorously applied. Incising the tumours when they are very tense and swollen relieves the pain at the point of origin, and the pain in the lymphatics gradually subsides under the antiseptic fomentations and the use of a sling.

Since the above was written a fifth case has come under observation, on July 11, 1908. The patient is the son of a farmer and has been dressing a flock of sheep for a disease of the mouth and lips. His father has never seen this disease of the sheep before in all his long experience as a flockmaster, and the veterinary surgeon is equally puzzled.

The case in the human patient is a mild one. There is one nodule on the knuckles, which is more irritable than painful; there is no inflammation of the lymphatics of the arm. The nodule has the same brawny hardness as in the other cases, but has no purple colouration in the centre. Up to the present the patient has not come up for inspection again; so, most probably, the swelling is gone.

